# Clinical Cases

**Published:** 1882-11

**Authors:** E. P. Gregory

**Affiliations:** Waterbury, Conn.


### CLINICAL BUREAU.

### CLINICAL CASES.

E. P. GREGORY, M. D., WATERBURY, CONN.

June 12th, 1882, was called about 6 P. M., to see C. E. B., machinist, æt. about twenty-five years. Stated that about an hour previous (5 P. M.) was taken with a severe chill, compelling him to leave work. Could obtain no characteristic symptoms, so gave Ipec. 200 D. to develop symptoms. June 14th, a second chill about 3.30 P. M. Complains of aching of legs; thirst during chill; cough during chill; Rhus 10 M. June 16th, chill again about 2 P. M., so violent as to shake the bed, with oppression of breathing; tongue clean, with thirst during all stages. Chinin sulph. 200 was given every four hours, with the result that he had a chill the *day following*, June 17th, at 5 P. M. The hour of the onset of the first chill, S. L. was sent. June 18th, chill lighter, anticipates one hour, ℞ S. L. June 20th, chill still lighter, ℞ S. L. Two lighter chills followed, and that was the end.

1880. M. M., machinist, applied for relief of a ganglia on sheath of flexor tendons of fourth finger, situated in palm of right hand. This ganglia was as large as a hickory nut, and, of course, interfered greatly with the usefulness of the hand. Consultations with other physicians led him to hope for relief by surgical means only, and the assurance that medicine would cure only shook his faith in the prescriber. A one-drachm vial No. 30 pellets, medicated with Ruta 1 M. Sig, two pills every night. I n three months the whole trouble was gone, and with it rheumatism of the right leg that troubled him quite a little.

Ruta g. rarely fails me in cases of ganglia, and it has cured not a few.

---

